# Mucopolysaccharidosis type II detection by Naïve Bayes Classifier: An example of patient classification for a rare disease using electronic medical records from the Canadian Primary Care Sentinel Surveillance Network

**DOI:** 10.1371/journal.pone.0209018

**Published:** 2018-12-19

**Authors:** Behrouz Ehsani-Moghaddam, John A. Queenan, Jennifer MacKenzie, Richard V. Birtwhistle

**Affiliations:** 1 Centre for Studies in Primary Care, Department of Family Medicine, Queen’s University, Kingston, Ontario, Canada; 2 McMaster University, Department of Pediatrics, Division of Genetics, Hamilton, Ontario, Canada; Zhejiang University, CHINA

## Abstract

Identifying patients with rare diseases associated with common symptoms is challenging. Hunter syndrome, or Mucopolysaccharidosis type II is a progressive rare disease caused by a deficiency in the activity of the lysosomal enzyme, iduronate 2-sulphatase. It is inherited in an X-linked manner resulting in males being significantly affected. Expression in females varies with the majority being unaffected although symptoms may emerge over time. We developed a Naïve Bayes classification (NBC) algorithm utilizing the clinical diagnosis and symptoms of patients contained within their de-identified and unstructured electronic medical records (EMR) extracted by the Canadian Primary Care Sentinel Surveillance Network (CPCSSN). To do so, we created a training dataset using published results in the scientific literature and from all MPS II symptoms and applied the training dataset and its independent features to compute the conditional posterior probabilities of having MPS II disease as a categorical dependent variable for 506497 male patients. The classifier identified 125 patients with the highest likelihood for having the disease and 18 features were selected to be necessary for forecasting. Next, a Recursive Backward Feature Elimination algorithm was employed, for optimal input features of the NBC model, using a k-fold Cross-Validation with 3 replicates. The accuracy of the final model was estimated by the Validation Set Approach technique and the bootstrap resampling. We also investigated that whether the NBC is as accurate as three other Bayesian networks. The Naïve Bayes Classifier appears to be an efficient algorithm in assisting physicians with the diagnosis of Hunter syndrome allowing optimal patient management.

## Introduction

The definition of what constitutes a rare disease varies in the medical literature [1, 2, 3]. For this study, a rare disease is defined as “a life threatening, seriously debilitating, or serious chronic condition that only affects no more than 5 in 10,000 people” [1]. The burden of rare diseases is high affecting as many as 1 in 12 Canadians and their families and has a significant impact on our healthcare and social systems, the workplace, the economy, and the collective social welfare [4]. People living with rare diseases encounter many challenges in obtaining appropriate and timely medical care. Among these challenges, delays in the diagnosis of the disease is an important and difficult one. According to a survey conducted from January–March 2015 on 491 Canadian patients with rare diseases, about two out of five respondents believed that their rare conditions were genetic, but almost none of them received any prenatal counselling or screening. They also experienced a significant delay and difficulty in receiving a correct diagnosis. While one-fourth received their diagnosis in less than three months, for one-third it took more than three years, and for one-fifth of respondents it took more than six years to obtain a correct diagnosis [4].

Compared to common diseases, the diagnosis of rare diseases is more complex, primarily because of limited awareness of the diseases and limitations in obtaining diagnostic testing. The consequence of delay in diagnosis or incorrect diagnosis has the potential to be catastrophic e.g., clinical worsening of the patient’s health in terms of physical, intellectual, psychological conditions, which sometimes even results in death of the patient; inappropriate treatment, inappropriate behavior, inadequate support from family members/society, and loss of confidence in the healthcare system [2]. Therefore, any technique that assists health practitioners in clinical assessment and making a diagnosis as early as possible has the potential to make a significant positive impact on the patient’s life.

Hunter syndrome or Mucopolysaccharidosis type II (MPS II) is an X-linked inherited disorder, due to mutations in *IDS* resulting in a deficiency of the lysosomal enzyme, iduronate 2-sulphatase [5, 6]. It has been estimated that about 0.6–1.3 in 100,000 male births are affected by MPS II [7]. Expression of the disease and the rate of progression are significantly variable and manifest at different ages. Some patients may develop mild physical problems and intellectual disabilities, while others may have more severe physical complications and mental disabilities. MPS II can affect many organ systems, including the nervous, cardiovascular, gastrointestinal, respiratory and musculoskeletal systems [7, 8, 9, 10]. Due to the variability of the initial symptoms and progressive nature of the disease, early diagnosis of MPS II is very challenging and often made after the obvious symptoms have developed which in its severe case, it will be when the patient is between 2 and 4 years old.

A Naïve Bayes Classifier (NBC) is a simple supervised machine learning technique and a conditional probability model based on Bayes’ theorem. NBC is “naïve” because it assumes that the features or variables in the model are independent of each other. Despite its naïve conditional and independence assumption and oversimplifying relationship among features, which is hardly true in the real world, the NBC is one of the most efficient and effective inductive learning algorithms for machine learning and data mining [11].

This study is the first part of a two-part project aimed at determining the practicality of the NBC and regression model in evaluating patients for MPS II disease. In the first part, we sought to evaluate the NBC algorithm as a simple method of identifying a “group” of patients with the highest likelihood of having MPS II as a rare disease with relatively common features using a “real” dataset. We also constructed a tree augmented Naïve-Bayes network (TAN), a Bayesian network augmented Naïve-Bayes network (BAN) and a Markov blanket Bayesian network (MBN) and compared the performance of these three Bayesian (belief) networks with that of NBC. In the second part, we used zero-inflated Poisson regression model to estimate the likelihood and risk of having MPS II disease for any “individual” patient using an “artificial” dataset simulated from patients who were previously classified by the NBC algorithm [12]. In the second part, more investigations were carried out to discover the underlying pattern of predictors and their contributions into the likelihood of having MPS II disease. The second part of this study can be considered as a complementary exploration that will make the disease forecasting possible for new patients and for those who their data and features were not used for training, test or validation of the NBC. These two parts have been reported into two separate papers mostly because of a massive amount of information, including the supplementary files and similarities and differences in techniques that we employed, which may cause confusion for readers.

## Methods

### Study population

The Canadian Primary Care Sentinel Surveillance Network (CPCSSN) is the first Canadian national multi-disease surveillance initiative based on the use of de-identified electronic medical records, which was funded by the Public Health Agency of Canada under a contribution agreement with the College of Family Physicians of Canada on behalf of 12 primary care research networks associated with the departments of Family Medicine across Canada. After approval of the project by the Health Sciences and Affiliated Teaching Hospitals Research Ethics Board (HSREB) at Queen’s University, a subset of CPCSSN’s database from 2016, which included all male patients (n = 506497) from networks in Calgary, Manitoba, Ontario and Newfoundland who had at least one visit to a primary care clinic in the past 24 months, was selected and their unstructured data were processed for subsequent steps. This definition of a practice population is according to Menec et al report, that most Canadian patients suffering from one or more chronic conditions have at least 1 primary care visit in 2 years [13].

### Data mining

CPCSSN has executed a framework that uses XML specification files that hierarchically define specific inclusion and exclusion text string criteria that, when applied to the original text fields (i.e., source from EMRs) it would improve the level and quality of coding in CPCSSN data. The framework is applied to diagnoses, medications, allergies, vaccines and lab results. There are specific algorithms for each specific diagnosis, which have been developed by having clinicians and other health providers to review the original text on matching strings that should be included in the algorithms. Structured data was created by text normalization including uppercasing letters, removing punctuation or unnecessary words and converting the original texts written by doctors to their closest ICD-9 definition (DiagnosisText_calc), followed by lemmatization and stemming for reducing word variations to simpler forms. For example, the following original texts converted to DiagnosisText_calc and then they were classified under Otitis: Chronic serous media/ Infective otitis externa (CHRONIC SEROUS OTITIS MEDIA = ICD-9 code 381.1) or other otitis externa (OTHER OTITIS EXTERNA = ICD-9 code 381.2), etc.

**Fig 1:**
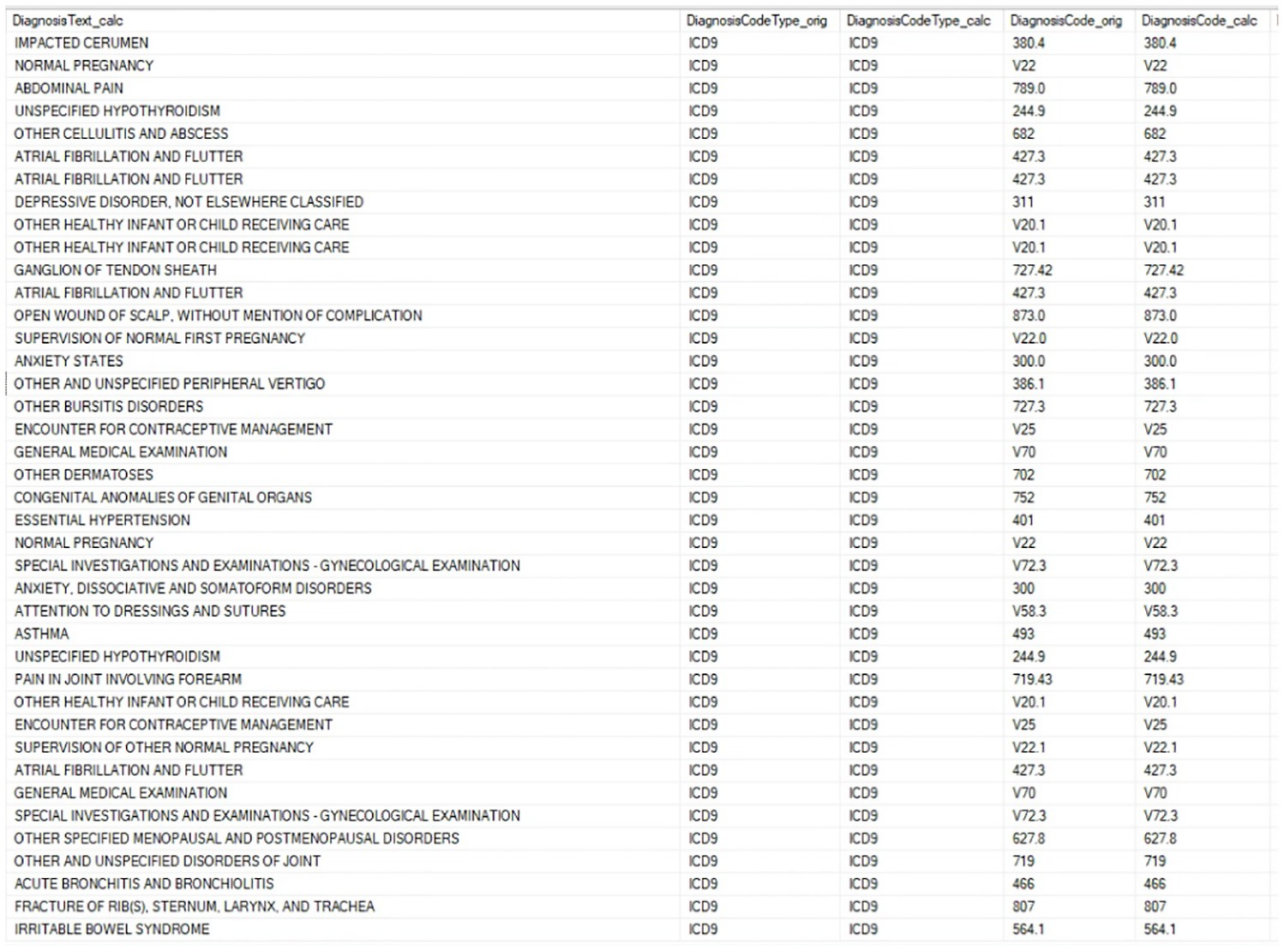
A screenshot of the billing table from SQL server containing unstructured data from patients.

**Fig 2:**
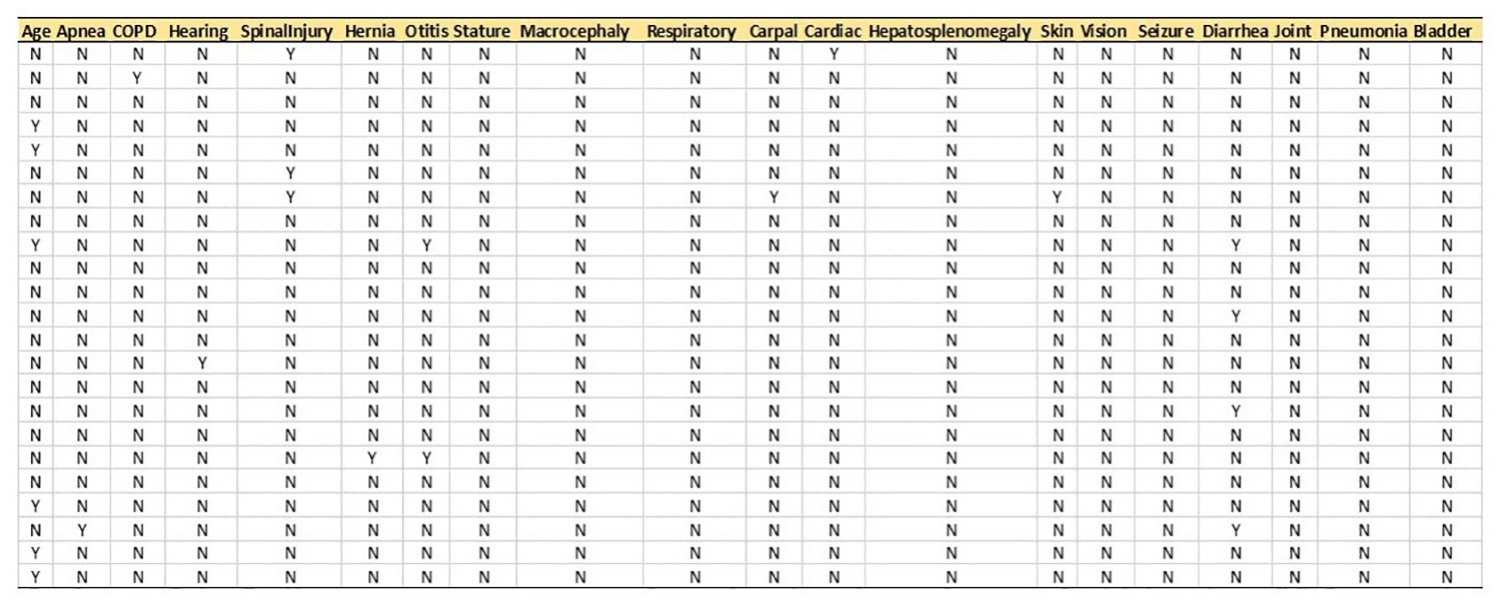
A screenshot of the MPS II dataset containing all symptoms from patients with dichotomous observations.

Prior to analysis, the structured data was sorted and cleaned. Cleaning included: removing duplicate records, detecting and modifying mismatched observations, identifying outliers and incorrect data and dropping unnecessary or irrelevant variables and appending encounter records of patients with their demographic records. Given a list of possible symptoms for MPS II, a text searching was conducted using billing, health condition and encounter diagnosis tables of patients for all signs and symptoms. Fig 1 shows a screenshot of part of the billing table in SQL server containing unstructured data from patients. Then, a dataset was created by extracting all the MPS II symptoms [7, 8, 9, 10] from patients and converting them to dichotomous observations (1 for the presence of symptom or 0 for otherwise) (Fig 2).

We then calculated an MPS index (MPSi) for each patient using the following multivariable equation:

$$\begin{array}{l} MPSi\;=\;\;[(0.917)\;\times \;Stature]\;\;+\;\;[(0.917)\;\times \;Macrocephaly]\;\;+\;\;[(0.290)\;\times \;Joint]\;\;+\;\;[(0.537)\;\times \;Apnea]\;\;+\\ \;[(0.536)\;\times \;COPD]\;\;+\;\;[(0.917)\;\times \;Hearing]\;\;+\;\;[(0.155)\;\times \;Vision]\;\;+\;\;[(0.153)\;\times \;Spinal]\;\;+\\ \;[(0.814)\;\times \;Hernia]\;\;+\;\;[(0.836)\;\times \;Otitis]\;\;+\;\;[(0.493)\;\times \;Respiratory]\;\;+\;\;[(0.123)\;\times \;Pneumonia]\;\;+\\ \;[(0.643)\;\times \;Carpal]\;\;+\;\;[(0.699)\;\times \;Cardiac]\;\;+\;\;[(0.863)\;\times \;Hepatosplenomegaly]\;\;+\;\;[(0.014)\;\times \;Bladder]\;\;+\\ \;[(0.274)\;\times \;Skin]\;\;+\;\;[(0.153)\;\times \;Seizure]\;\;+\;\;[(0.521)\;\times \;Diarrhea]\; \end{array}$$ (1)

Where numbers inside parentheses represent the approximate frequency of symptoms associated with MPS II disease estimated from previous studies on a population with high diversity similar to Canadian population [9, 10]. For example, for a patient who has joint pain, COPD and diarrhea and no other MPS II symptoms, the index is:

$$\begin{array}{ll} MPSi & =\;[(0.29)\;\times \;Joint]\;+\;[(0.536)\;\times \;COPD]\;+\;[(0.521)\;\times \;Diarrhea]\\ & =\;0.29\;+\;0.536\;+\;0.521\\ & =\;1.347 \end{array}$$

The purpose of making MPSi parameter was to select patients with the lowest number of symptoms for succeeding training dataset.

### NBC creation

A Naïve Bayes Classifier is a set of supervised learning algorithms based on conditional probability derived from Bayes theorem:

$$P\left(y|x_{1,}x_{2,\ldots ,}x_{n}\right)=\frac{P\left(x_{1,}x_{2,}\ldots ,x_{n}|y_{j}\right)P\left(y\right)}{P\left(x_{1},\;x_{2}\ldots ,x_{n}\right)},$$ (2)

Where *x*_*i*_ is a categorical and/or numerical predictor (independent variable), *y*_*j*_ is a class vector or the categorical levels of dependent variable (outcomes), *p(y*_*j*_ *| x*_*1*_, *x*_*2*_, *…*, *x*_*n*_*)* and *p(x*_*1*_, *x*_*2*_, *…*, *x*_*n*_ *| y*_*j*_*)* are the posterior and prior probabilities of class membership, respectively.

By conditional independence assumption, the joint probability model can be expressed as:

$$P\left(y|x_{1,}x_{2,\ldots ,}x_{n}\right)\;\propto \;P\left(y\right)\overset{n}{\underset{i=1}{\prod }}P\left(x_{i}|y\right),$$ (3)

By choosing the highest posterior probability, which is called Maximum A Posteriori or MAP decision rule, the NBC algorithm can classify a new case *x*_*i*_ with a class level *y*_*j*_ using the following formula:

$$\hat{y}=\text{arg}\;maxP\left(y\right)\overset{n}{\underset{i=1}{\prod }}P\left(x_{i}|y\right)$$ (4)

**Fig 3:**
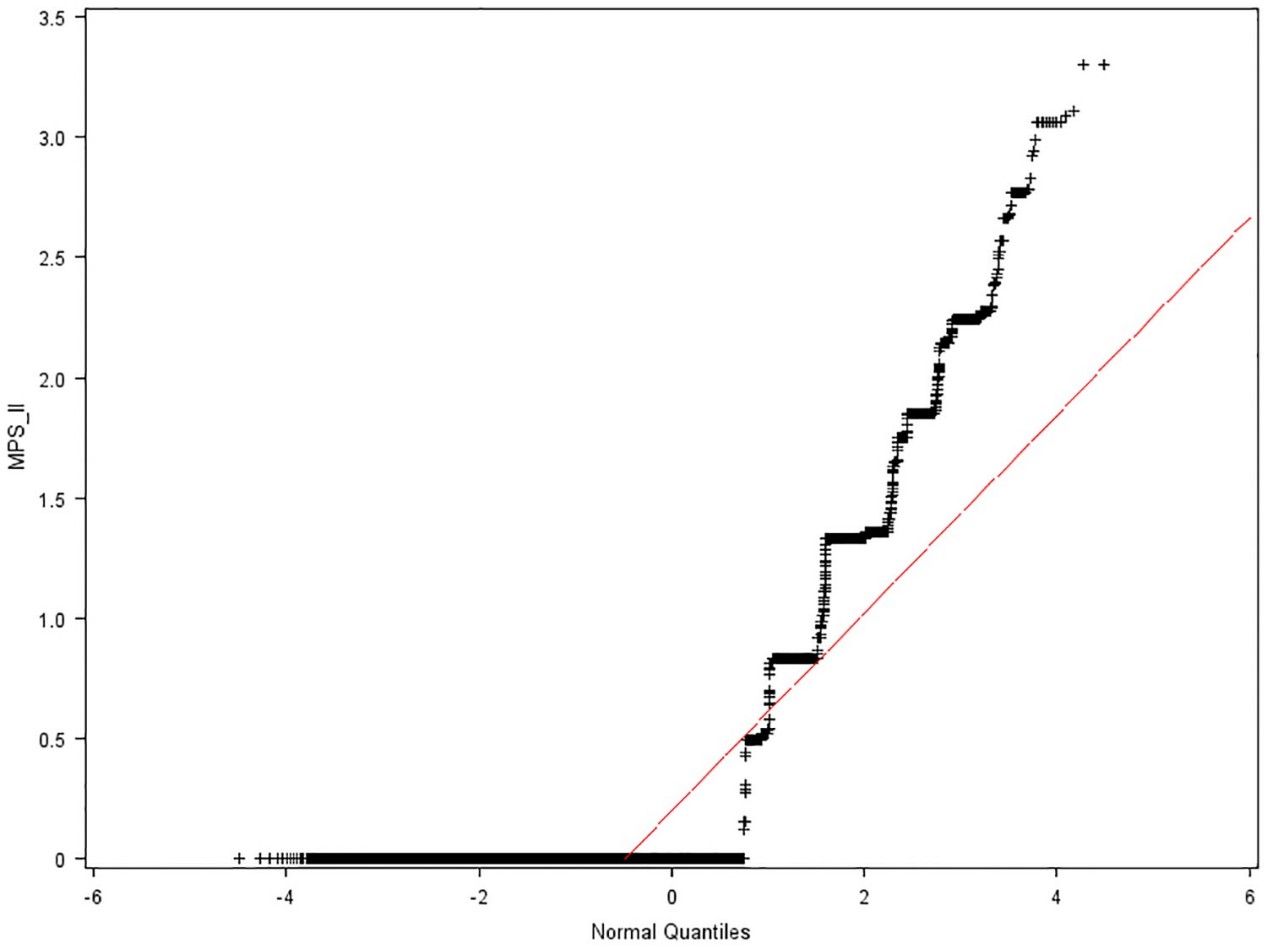
Normal Q-Q plot of MPS II index from patients 21 years old or younger. Red line represents a distribution reference line with μ_o_ equal to the sample mean for a normal distribution.

**Fig 4:**
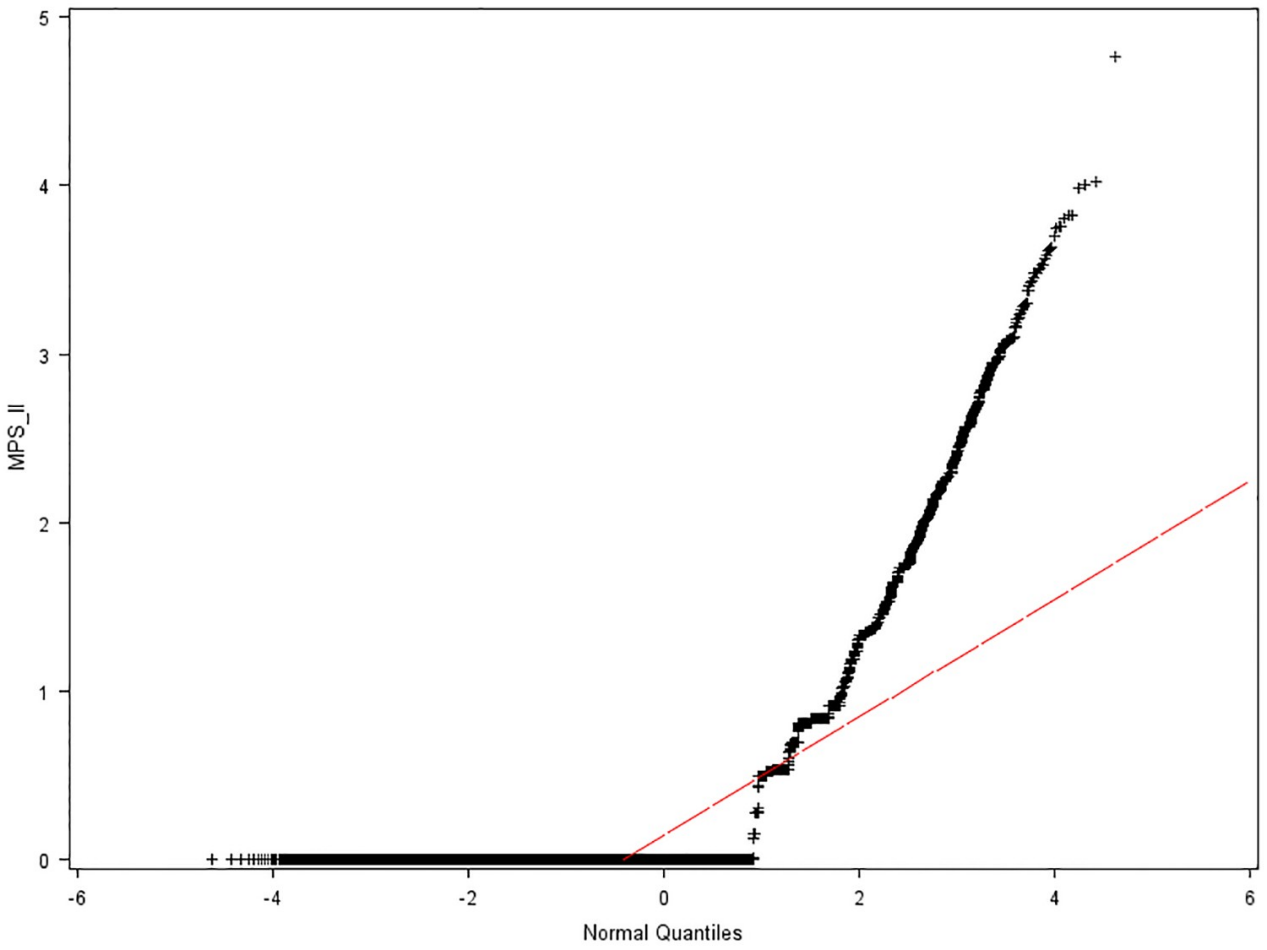
Normal Q-Q plot of MPS II index from patients older than 21. Red line represents a distribution reference line with μ_o_ equal to the sample mean for a normal distribution.

Where $\hat{y\;}$ is the estimated class category of outcome. In Bayesian statistical inference, prior probabilities are based on previous experience or available information, which in the NBC refers to training data. Within the constructed CPCSSN’s male population dataset, 971 patients without MPS II, who had the lowest MPSi were selected randomly using Q-Q plots (Figs 3 and 4) and their normal percentiles from the following two age groups.

Y: 21 years old ≥ age (Fig 3);
N: 21 years < age (Fig 4).

The original patient’s age format was numeric and continuous; nevertheless, in the training data set, which was constructed using information from previous study [9], age was recorded as categorical observations. To avoid any sampling bias because of lack of enough patients in some age groups, the age format was converted from continuous to binary, so that the age classes become more uniformed. Additionally, MPS II is a progressive disease, the older patients (over 21 years old) who have developed more symptoms caused by other diseases, might be falsely classified as MPS II positive. On the other hand, for some young patients with very few symptoms, the NBC algorithm, which operates on features, may fail to detect these patients. Therefore, classification using two age groups is more accurate.

These 971 actual patients without MPS II together with a group of 73 patients with MPS II, which was created artificially by frequency distribution table for dichotomous features using available information from previously published results [9, 10], produced our training dataset for further estimation of probabilities of having disease for each person. The resulting training dataset contained 1044 patients (observations) and their corresponding 20 attributes (columns), i.e. 19 symptoms and 1 variable as patient’s age. The final training sample size was determined using plotting learning curves [14, 15]. Next, we applied the training dataset and its independent features (including all available symptoms) to compute the conditional posterior probabilities of having MPS II disease as a categorical dependent variable for all CPCSSN’s male patients (n = 505526).

### NBC performance evaluation

In an NBC algorithm, we assume conditional independence between the predictive features. In order to test this assumption, and eliminate highly correlated features, which may cause multicollinearity problems, a phi coefficient (ɸ) was computed to estimate the degree of relationship among features in the model [16].

We used two different techniques to select features, a wrapper method and a filter method [17, 18]. The wrapper included the recursive backward feature selection and filter method included feature importance from ROC (receiver operating characteristic) analysis. Recursive Backward Feature Elimination algorithm was employed for optimal input features of the NBC model. The dataset was partitioned into 10 subsets of data, in which 9 parts were used for training and the remaining subset was used for testing. The process of training and testing was repeated for the remaining 9 parts. The algorithm was configured to explore all possible subsets of the features. Positive predictive value of each individual feature was also used for optimal input features of the model. The positive predictive value cut-off point was set to 0.15. The actual selection of this cut-off point was arbitrary, but was partly based on information from accuracy parameters and the fact that features with the lowest PPV have the lowest contribution to the model and should be removed from the model; however, the elimination should be minimum, so that outliers (patients who have the features with low frequency) remain in the training dataset.

**Fig 5:**
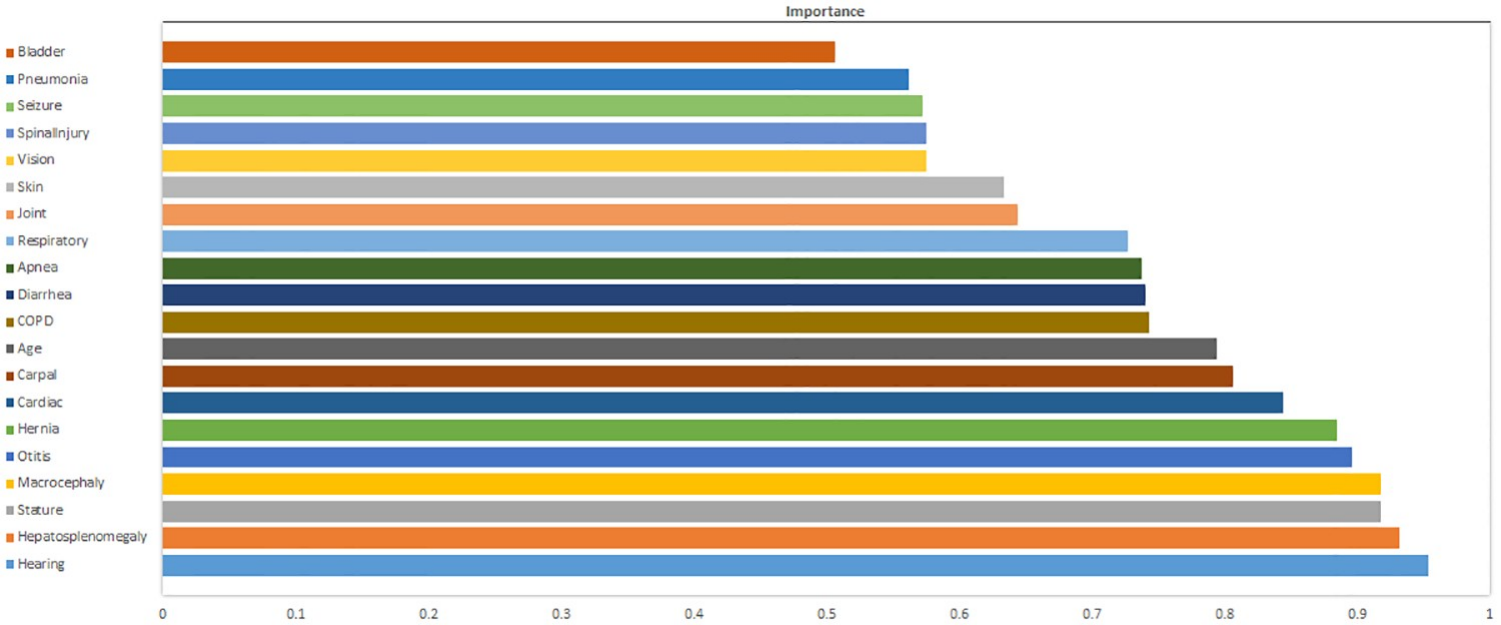
The importance of features for MPS II disease forecasting by the NBC algorithm estimated using a ROC curve analysis conducted for each attribute.

As we mentioned earlier, we also used a filter approach to calculate the overall importance of each feature by analyzing ROC individual on each feature using the method described by Kuhn (2008) and Kuhn and Johnson (2016) [17, 18]. The ROC curve represents the true positive rate (sensitivity) as a function of the corresponding false positive rate. The area under the ROC curve is a measure that indicates an overall estimation of the performance of a classifier. A series of cutoffs was applied to the feature data to predict the MPS II class membership. The sensitivity and specificity were calculated for each cutoff and using trapezoidal rule the area under the ROC curve (AUC) was computed by using the predictor as input to the ROC curve. If the predictor could separate the classes of MPS II, that was an indicator that the feature was an important predictor (in terms of class separation) and this was captured in the corresponding AUC statistic. The maximum AUC was automatically computed by the *caret* package of R program and considered as the measure of variable importance. We used Microsoft Excel to plot the importance for each feature (Fig 5).

To measure the uncertainty associated with our statistical learning technique, resulting from over-training of the NBC model and in order to estimate the accuracy of the final model, bootstrap resampling [19, 20] was performed by taking randomly 1000 samples. The model performance was also evaluated by the Validation Set Approach technique [19, 21], which was carried out by partitioning randomly 70% of the dataset for training and the rest for evaluating the model performance. The result of the analysis of this section was expressed by accuracy, Kappa and other associated statistics that are often calculated from a confusion matrix for a binary classifier.

### Comparison between the NBC and other Bayesian networks

A Bayesian (belief) network is a directed acyclic graph (DAG) representation of the joint probability distribution of a set of random variables, in which each node in the graph corresponds to domain variables and the arcs between nodes represent probabilistic dependencies among the corresponding variables [22]. There are two assumptions of the NBC model: 1. all features are conditionally independent of each other; 2. all features directly depend on the dependent variable. In a tree augmented Naïve-Bayes network (TAN) by relaxing the first assumption of the NBC, we allow arcs between the children of the classification node [23]. In a Bayesian network augmented Naïve-Bayes network (BAN), all feature nodes are direct children of the classification node, but a complete Bayesian network is constructed between the child nodes [24]. Lastly, in a Markov blanket Bayesian network (MBN), after the Bayesian network is fully constructed and prior to using the network for classification, all nodes outside the Markov blanket are deleted [25].

These three models are more complex than the NBC. A model that is not adequately complex may fail to detect fully the signal in a data, causing underfitting. On the other hand, the models that are too complex might fit both noise and signal, causing overfitting. To test whether the NBC is sufficiently complex, we constructed these three Bayesian networks (TAN, BAN and MBN) using all 20 attributes from the validation dataset and their performances were compared with that of the NBC. Independence test between the features and the target variable for all these networks was carried out using Chi-square test. The accuracy parameters, misclassification rate, average and sum of squared error of four networks were used for performance comparison. The result of this part of the study was summarized into a table and illustrated in four graphs.

### Statistical software

Data extraction from SQL server and data mining including data cleaning procedure were carried out by SAS (version 9.4 TS). Naïve Bayes classification and its model performance assessment including resampling and bootstrap procedures and phi coefficient analysis were carried out by *e1071*, *caret*, *pROC*, *SDMTools*, *ROCR* and *klar* packages of R program (version 3.4.3). SAS Enterprise Miner (version 14.3) carried out performance comparison between the NBC and other networks.

## Results

### NBC creation

In this study, we analyzed 505526 male patients. The NBC algorithm identified 125 patients with the highest probability for having MPS-II disease. The prevalence of the disease among male is estimated to be 0.025%. The frequent occurrence of various symptoms, symptom combinations and their relevant occurrences for those who were identified as potential patients with MPS II disease have been shown in Table 1. In the table, only those symptom combinations with the incidence of 1.6% or higher have been presented. Hearing loss and Otitis with 58 cases and with 11.9% incidence rate were the most common symptoms followed by COPD with 47 cases and with 9.6% incidence rate and Hernia and Cardiac each with 45 cases and with 9.2% incidence rate. According to this table, combination of Hearing + Otitis + Hernia + Respiratory problem with the incidence of 6.4% was the highest among patients, followed by the combination of Hearing + Otitis + Hernia + Cardiac; Hearing + Otitis + COPD + Cardiac + Diarrhea and the combination of Hearing + Otitis + COPD + Cardiac + Respiratory problem, each with 2.4% incidence rate.

**Table 1:** Symptom combinations for potential patients diagnosed with MPS II disease by NBC algorithm. Only the combinations with 1.6% incidence or higher have been presented here.

	Hearing	Otitis	COPD	Hernia	Cardiac	Respiratory	Diarrhea	Apnea	Carpal	Spinal Injury	Skin	Hepatosplenomegaly	Seizure	Joint	Stature	%
	x	x		x		x										6.4
	x	x		x	x											2.4
	x	x	x		x		x									2.4
	x	x	x		x	x										2.4
	x	x		x					x							1.6
	x	x		x	x	x										1.6
	x	x					x			x	x					1.6
	x			x	x					x						1.6
	x		x		x		x				x					1.6
		x	x	x	x				x							1.6
		x	x	x	x	x										1.6
		x	x	x	x					x						1.6
	x	x				x	x	x								1.6
					x			x	x			x				1.6
			x		x		x	x	x							1.6
			x	x	x	x		x								1.6
**Total**	58	58	47	45	45	42	39	38	35	33	19	13	11	4	2	
**(%)**	11.9	11.9	9.6	9.2	9.2	8.6	8.0	7.8	7.2	6.7	3.9	2.7	2.2	0.8	0.4	100

### NBC performance evaluation

Phi coefficient correlation analysis using all features in the NBC model did not show any substantial correlation among features, indicating that there was no multicollinearity among predictors in our model and that the assumption of independency among attributes was valid.

Table 2 shows all features and their names and corresponding symptoms entered into the NBC algorithm and those remained in the model (in bold). Out of 20 features entered the model, 18 attributes were selected and remained in the model using the Recursive Backward Feature Elimination algorithm and by positive predictive value of each individual feature (Table 3). The importance of features was also estimated using a ROC analysis conducted for each attribute and the results were presented in Fig 5 in a scale from 0 to 1. According to this plot, Hearing (loss) with importance score 0.95 was the most important feature in the dataset for MPS II forecasting, followed by Hepatosplenomegaly with importance score 0.93, Macrocephaly and Stature each with importance score 0.92. According to Fig 5 and Table 3, Bladder and Pneumonia were features with the least importance, and the least PPV and therefore, they were eliminated from the final model.

**Table 2:** Features and their associated symptoms in MPS II disease. The remained features in the final NBC model are show in bold.

Feature name	Symptom/Description
**Stature**	Short stature, contracture, coarse facial features, congenital Musculoskeletal
**Joint**	Joint pain, joint stiffness
**Apnea**	Sleep apnea
**COPD**	COPD, airway obstruction
**Hearing**	Progressive hearing loss
**Spinal injury**	Spinal cord injury, spinal stenosis, compression, dysostosis, congenital musculoskeletal
**Hernia**	Umbilical hernia, inguinal hernia
**Otitis**	Chronic ear infections, AOM, otitis
**Respiratory**	Respiratory infection
**Carpal**	Carpel tunnel syndrome
**Cardiac**	Cardiac disease, heart valve problem, cardiac problem, ventricular hypertrophy
**Hepatosplenomegaly**	Hepatosplenomegaly, hepatomegaly, enlarged liver, splenomegaly, enlarged spleen
**Skin**	Pebbly skin lesion, thickened skin
**Seizure**	Seizure
**Diarrhea**	Diarrhea
**Age**	Patient’s age: (1 for younger than 21or 0 for otherwise)
**Macrocephaly**	Macrocephaly, enlarged head
**Vision**	Vision problem, reduced vision or visual problems
Pneumonia	Recurrent pneumonia
Bladder	Bladder obstruction

**Table 3:** Accuracy and Kappa values of features in the NBC model derived from Recursive Backward Feature Elimination algorithm and their positive predictive value.

Variables	Accuracy	Kappa	Accuracy SD	Kappa SD	PPV*
**Age**	0.9847	0.8872	0.01	0.08	0.93
**Apnea**	0.9863	0.8883	0.01	0.09	0.49
**COPD**	0.9898	0.9113	0.01	0.08	0.51
**Hearing**	0.9904	0.9179	0.01	0.06	0.92
**Spinal Injury**	0.9904	0.9179	0.01	0.06	0.15
**Hernia**	0.9904	0.9179	0.01	0.06	0.78
**Otitis**	0.9885	0.8986	0.01	0.09	0.84
**Stature**	0.9885	0.8986	0.01	0.09	0.84
**Macrocephaly**	0.9885	0.8986	0.01	0.09	0.84
**Respiratory**	0.9885	0.8986	0.01	0.09	0.49
**Carpal**	0.9885	0.8986	0.01	0.09	0.62
**Cardiac**	0.9885	0.8986	0.01	0.09	0.70
**Hepatosplenomegaly**	0.9885	0.8986	0.01	0.09	0.86
**Skin**	0.9885	0.8986	0.01	0.09	0.27
**Vision**	0.9885	0.8986	0.01	0.09	0.15
**Seizure**	0.9885	0.8986	0.01	0.09	0.15
**Diarrhea**	0.9885	0.8986	0.01	0.09	0.52
**Joint**	0.9885	0.8986	0.01	0.09	0.29
**Pneumonia**	0.9885	0.8986	0.01	0.09	0.12
**Bladder**	0.9885	0.8986	0.01	0.09	0.01

* Positive predictive value

Table 4 reveals the result of performance evaluation of the NBC model in a 2 × 2 contingency table using the bootstrap resampling and the Validation Set Approach technique on test dataset. According to the table, there were 376248 (97.97%) correct classifications (357251 for “No” and 18997 for “Yes”, along the diagonal) and 7815 (2.03%) incorrect classifications (0 for “No” and 7815 for “Yes”, along the vertical). The overall accuracy, Kappa, sensitivity and specificity of the predictive model for test dataset were 0.99, 0.91, 0.84 and 1, respectively.

**Table 4:** The NBC model performance. The 2 × 2 contingency tables displays the performance evaluation using the bootstrapped resampling (n = 1000) and the Validation Set Approach technique on test dataset. Accuracy was used to select the optimal model by the largest value.

	Actual		
Predicted	No	Yes	Row Total
**No**	357251	7815	365066
**Yes**	0	18997	18997
**Column Total**	357251	26992	384063
**Accuracy**	0.99		
Kappa*	0.91		
Sensitivity*	0.84		
Specificity*	1.0		

* Estimated by the Validation Set Approach technique

**Fig 6:**
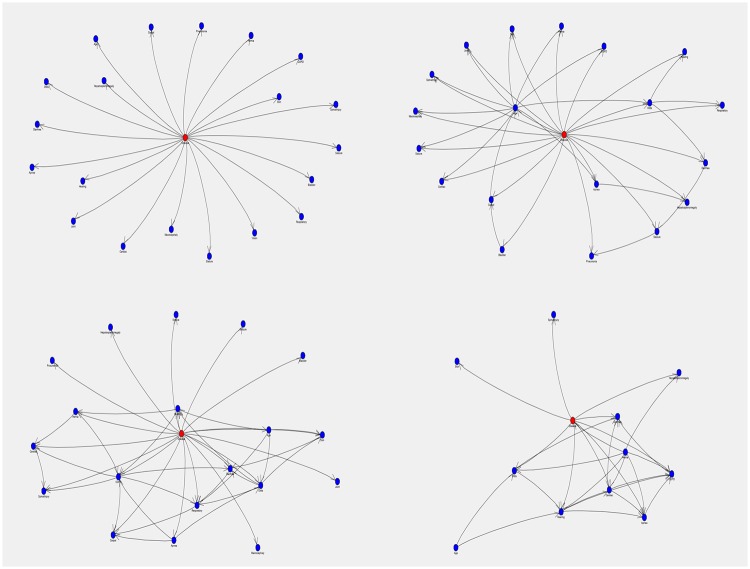
Bayesian network classifiers. Top left: NBC = Naïve Bayes classifier; top right: TAN = Tree augmented Naïve-Bayes network; bottom left: BAN = Bayesian network augmented Naïve-Bayes network; bottom right: MBN = Markov blanket Bayesian network. Red circles are target variable (MPS II disease) and dark blue circles are features.

The accuracy parameters, misclassification rate, average and sum of squared error of the NBC and three other Bayesian networks (TAN, BAN and MBN) are shown in Table 5. According to this table, the MBN was the model with the lowest ASE (0), the lowest SSE (62.720) and with perfect accuracy (1.0). On the contrary, the false negative rate of the model was the highest (21 cases) and the sensitivity was the lowest among other networks (0.447). In this evaluation, the performance of BAN model appeared to be slightly better than other networks followed by TAN, NBC and MBN. The Bayesian network structure from validation dataset for each network has been illustrated in Fig 6.

**Table 5:** Performance comparison of Bayesian network classifiers using validation dataset.

Classifier	FN	TN	FP	TP	Accuracy	Sensitivity	Specificity	MR	ASE	SSE	ROC Index
**NBC**	0	147714	4198	38	0.972	1.000	0.972	0.028	0.022	6600.840	1.000
**TAN**	0	148099	3813	38	0.975	1.000	0.975	0.025	0.019	5829.130	1.000
**BAN**	0	148587	3325	38	0.978	1.000	0.978	0.022	0.016	4836.650	1.000
**MBN**	21	151890	22	17	1.000	0.447	1.000	0.000	0.000	62.720	0.999

NBC = Naïve Bayes Classifier; TAN = Tree augmented Naïve-Bayes network; BAN = Bayesian network augmented Naïve-Bayes network; MBN = Markov blanket Bayesian Network; FN = false negative; TN = true negative; FP = false positive; TP = true positive; MR = misclassification rate; ASE = average squared error; SSE = sum of squared error.

## Discussion

A Naïve Bayes classifier model was developed using the clinical diagnosis and symptoms of patients to estimate MPS II likelihood in patients, and it was evaluated for its accuracy in forecasting the disease by the Validation Set Approach and the bootstrap resampling techniques. Based on the results of performance evaluation on test dataset, presented in Table 4, the NBC model has an accuracy level about 0.99 to identify MPS II disease. There were also high rates of sensitivity, specificity and a Kappa value with almost perfect agreement [21] for the classifier. The Naïve Bayes appears to be an efficient classifier that may help physicians with the diagnosis of Hunter syndrome. This is only the first step in determining whether this technique is of value however. The database used is de-identified and we have no idea whether any of these patients have MPS II. In order to determine this the next step will be to contact the patient’s primary care practitioner and patient or patient’s family to ask if they would undergo testing to determine a true diagnosis. This will require careful planning and discussion of the ethical and practical implications of this approach.

In spite of oversimplifying relationship among features, the performance of NBC is very good. It is believed that, the reason for such performance is the fact that, it is the distribution of dependencies among all features over classes that influences the classification of naive Bayes, not just the dependencies themselves [11]. Another advantage of the NBC technique is its ability to process complex queries or when the dimensionality of the dataset is very high [26], nevertheless, higher dimensionality causes an outcome classification become extremely computationally expensive.

Another reason for choosing the NBC for our study was the interpretability of the model. In machine learning technique, sometimes achieving high accuracy metrics alone is not sufficient to evaluate the model performance. In many cases, the model also has to give an explanation how it came to the answer, because an accurate prediction only partially solves the original problem [27]. According to Miller (2017), interpretability during the computation is how well a human could understand the decisions in the given context or the degree to which an observer can understand the cause of a decision [28]. The higher the interpretability of a model, the easier it is to understand why a certain decision has been made. There are three criteria that are equally important for interpretability and should be part of a decision-making algorithm: simplicity, generality, and coherence [28]. A model has better interpretability than another model, if its decision is easier to comprehend for a human than decisions from the second model. Simple statistical models such as linear regression model, the decision tree and the NBC are considered as interpretable models. The NBC is an interpretable model, because of the independence assumption, which makes it clear, how much influence each feature has towards a certain class prediction. Complex models such as neural networks, on the other hand, are considered as black boxes [27]. Because, for many people the part between the input variables and the response from these models is incomprehensible. When using machine learning for medical diagnosis, for example, predictions cannot be utilized without truly understanding the algorithm, as it has the potential to have a tragic consequence. Accordingly, a practitioner may wish to choose a less accurate model for content recommendation that does not place high importance in features, even though exploiting such features increases the accuracy of the model in cross validation [29].

The two assumptions of the NBC algorithm significantly reduces the complexity of the Bayesian classifier including the computation of the posterior, because we only need the prior probability of the class and the conditional probabilities of each attribute given the class. For example in Fig 6 (top left), every node (symptom) is directed towards the target node (disease), assuming each node is independent from one another. In addition, after learning from data, the NBC never comprehends more components than there are in the data, promising that inference will be manageable [30]. Unlike the NBC, other Bayesian networks provide a more complex method of interdependency among features (Fig 6) in which having arcs between the children of the classification node is allowed, consequently, structures that are more complex can arise. In the BAN, for instance, the algorithm takes a form of the network that is extended to incorporate local high or low-level nodes; as a result, a hierarchy between nodes is born [31]. In Bayesian network, the direction of links identifies the conditional dependency of attributes, and those non-descendants, which have no link, are considered conditionally independent. This indicates that, the TAN, BAN and MBN networks combine interdependency relations with naïve Bayesian reasoning. These network models present a remarkable computational challenge. Structure learning about them is NP-hard in the general case [32], as is the inference [33] and when there are too many features; the network structure can be difficult to interpret. In contrary, the NBC is simpler, interpretable and more intuitive model. Overall, the result of the performance comparison between the NBC and other Bayesian networks (Table 5) supports the idea that the efficiency can be obtained under the independence assumption of the NBC model without losing a major amount of accuracy.

In this study, we also selected the NBC over some other popular classifiers such as Decision Trees and Support Vector Machine (SVM). There are some pros and cons for each algorithm, but the overall efficacy of the algorithm strongly depends on the size and structure of the dataset. For example, similar to NBC, Decision Trees are simple to understand and interpret [34, 35] and unlike the NBC, they easily handle interactions between features. However, since they use the divide and conquer method, they are more likely to perform well if a few extremely relevant features exist, but less productive if many complex interactions are present [36]. They are also prone to overfitting. Teaching a decision tree to recognize rare events such as rare diseases is very difficult, because rare events often are pruned out and the resulting tree will misclassify those important events. SVM are highly accurate, and similar to the NBC when there is a very high-dimensional space, they work very well. However, they are memory-intensive and unlike the NBC, their results are hard to interpret, and difficult to run and tune the algorithm. Another advantage of the NBC over these two classifiers is that, it does not require a large amount of data for training. This advantage was highly important for our study, because our training data from MPS II patients was small. Unlike both Decision Trees and SVM, the NBC is not sensitive to irrelevant features or noise. Moreover, in the estimation of the occurrence of a disease such as MPS II and using some features including symptoms, although the statement of independency among attributes is unrealistic, nonetheless, the assumption is very convenient and helpful, because in most cases such as in our study, we do not know the exact relationship or pattern among symptoms.

Variable importance, which was employed in this study and estimated based on the reduction of features accuracy when the feature of interest was removed, is one way to reduce the data dimension. In our study, the positive predictive value of each individual feature and the area under the ROC as the measure of variable importance on a scale 0–1 were used to select features, which resulted in the remaining of 18 variables in the final NBC model.

In addition to machine learning approach, there are other techniques that can be employed to identify rare diseases in a population. Among these techniques, Human Phenotype Ontology and Leveraging Collaborative Filtering are two relatively new and well-established methods, which both are based on concept recognition and natural language processing for extracting ontology terms from patients’ phenotypic information available in EMR with the intention of leveraging structured knowledge from unstructured data [37, 38].

Our study has several limitations. Firstly, there might be a misclassification as a result of the influence of patient’s age on the development of symptoms and its impact on subsequent outcome. Since, MPS II is a progressive disease that the age at onset of symptom is very decisive variable in the characteristic feature development, therefore, it is rational to expect that the older patients who have developed more symptoms caused by other diseases, may have been falsely classified as MPS II positive (type I error or false positive). On the other hand, some young patients, who truly have the disease, but they have zero or very few symptoms, the NBC algorithm, which operates on features, may fail to detect these patients (type II error or false negative). One conceivable solution to overcome such misclassification for progressive diseases such as MPS II is using different NBC algorithms for different age groups. Secondly, some bias in making data training for patients with positive outcome possibly existed. The bias caused by using different population and their incidence rates of MPS II symptoms for our Canadian populations. Thirdly, in our study we did not compare the efficacy of NBC with other non-Bayesian machine learning techniques. This study was a stepping-stone to a bigger and more comprehensive assessment of machine learning for rare disease prediction. In future studies, we will compare the efficacy of the NBC with other classifiers by incorporating other machine learning algorithms to our study. Fourthly, our inability to confirm the positive cases estimated by the NBC model with the gold standard test, which is an absence or reduction of Iduronate 2-sulfatase (I2S) enzyme activity in white cells, fibroblasts, or plasma [39]. However, using the NBC model presented here, one can make a short list of patients with the highest likelihood for having the disease. Given a short list of positive cases obtained by the NBC model, it would be more feasible for health care practitioners to request the I2S enzyme test to confirm or disprove the diagnosis. Consequently, the expensive and apprehensive gold standard test is reserved for the patients who had a positive result in a prescreening evaluation of the NBC model. However to better determine this, the next study will require those patients identified as having a higher probability of MPS II to be approached to confirm the diagnosis. It is likely that many of those will not have the disease so the ethics of approaching patients and telling them they may have a rare genetic disorder must be carefully considered. Data analysts who have access to a large volume of patient data and are able to employ some predictive modeling such as the Naïve Bayes classifier, have a precious opportunity to contribute their insights into the efficient identification of patients with rare diseases such as MPS II, as a result, may have a positive impact on disease diagnosis and potential earlier treatment.

## Conclusions

In this study, a predictive model for the MPS II disease was developed using the Naïve Bayes Classifier and CPCSSN database collected from male patients in Canada. The classifier identified 125 patients with the highest likelihood for having the disease and 18 features were considered to be necessary for risk prediction. It can be concluded that the Naïve Bayes Classifier is an efficient algorithm in assisting physician with the diagnosis of MPS II.

## References

[1] Supporting cutting-edge rare disease research. Canadian Institutes of Health Research. 2018; http://www.cihr-irsc.gc.ca/e/49695.html.

[2] Europian Organization for Rare Diseases. Rare Diseases: Understanding This Public Health Priority. 2005; https://www.eurordis.org/IMG/pdf/princeps_document-EN.pdf.

[3] U.S. Department of Health and Human Services. Orphan Drug Act. 2018; https://www.fda.gov/RegulatoryInformation/LawsEnforcedbyFDA/SignificantAmendmentstotheFDCAct/OrphanDrugAct/default.htm

[4] Canada’s Rare Disease Strategy. The Canadian Organization for Rare Disorders. 2017; http://www.raredisorders.ca//content/uploads/CORD_Canada_RD_Strategy_22May15.pdf

[5] Bach G, Eisenberg FJ, Cantz M, Neufeld EF. The defect in the Hunter syndrome: deficiency of sulfoiduronate sulfatase. Proc Natl Acad Sci USA. 1973; 70: 2134–2138. 4269173 10.1073/pnas.70.7.2134 PMC433682

[6] A Guide to Understanding Mucopolysaccharidosis (MPS) II. The Canadian Society for Mucopolysaccharide and Related Diseases Inc. 2017; https://www.mpssociety.ca/wp-content/uploads/2017/04/MPSIIBookletEnglish.pdf

[7] Schwartz I, Ribeiro MG, Mota JG, Toralles MBP, Correia P, Horovitz D. A clinical study of 77 patients with mucopolysaccharidosis type II. Acta Pædactrica. 2007; 96: 63–70. 10.1111/j.1651-2227.2007.00212.x 17391446

[8] Martin R, Beck M, Eng C, Giugliani R, Harmatz P, Muñoz V. Recognition and diagnosis of mucopolysaccharidosis II (Hunter Syndrome). Pediatrics. 2008; 121: 377–386. 10.1542/peds.2007-1350 18245410

[9] Needham M, Packman W, Quinn N, Rappoport M, Aoki C, Bostrom A, Cordova M, Macias S, Morgan C, Packman S. Health-Related Quality of Life in Patients with MPS II. J Genet Counsel. 2015; 24: 635–644. 10.1007/s10897-014-9791-7 25395377

[10] Mucopolysaccharidosis type II. Genetics Home Reference. 2016; https://rarediseases.info.nih.gov/diseases/6675/mucopolysaccharidosis-type-ii

[11] Zhang H. The Optimality of Naive Bayes. Proceedings of the Seventeenth International Florida Artificial Intelligence Research Society Conference. 2004; Miami Beach, Florida, USA.

[12] Ehsani-Moghaddam B, Queenan JA, MacKenzie J, Birtwhistle RV. Zero-inflated Poisson regression and Factor analysis of Mucopolysacharidosis type II using Electronic medical records from the Canadian Primary Care Sentinel Surveillance Network. 2018; submitted for publication. 10.1371/journal.pone.0209018 PMC6300265 30566525

[13] Menec V, Black C, Roos NP, Bogdanovic B, Reid R. Defining practice populations for primary care: Methods and issues. Manitoba Centre for Health Policy and Evaluation. 2000; http://mchp-appserv.cpe.umanitoba.ca/reference/roster.pdf

[14] Figueroa RL, Zeng-Treitler Q, Kandula S, Ngo LH. Predicting sample size required for classification performance BMC Med. Inform. Decis. Mak. 2012; 12: 8 10.1186/1472-6947-12-8 22336388 PMC3307431

[15] Beleites C, Neugebauer U, Bocklitzc T, Krafft C, Poppa J. Sample size planning for classification models. Analytica Chimica Acta. 2013; 760: 25–33. 10.1016/j.aca.2012.11.007 23265730

[16] Muir SW, Berg K, Chesworth B, Klar N, Speechley M. Balance impairment as a risk factor for falls in community-dwelling older adults who are high functioning: a prospective study. Phys Ther. 2010; 90:338–47. 10.2522/ptj.20090163 20056721

[17] Kuhn M. Building predictive models in R using the caret package. Journal of Statistical Software. 2008; 28(5): 1–26. 10.18637/jss.v028.i0527774042

[18] Kuhn M. & Johnson K. Applied Predictive Modeling, Springer New York 2016; 19: 491–500.

[19] James G, Witten D, Hastie T, Tibshirani R. An Introduction to Statistical Learning: with Applications in R. Springer 2013; 4: 175–194.

[20] Efron B, Tibshirani R J. An introduction to the bootstrap. 1993; NewYork: Chapman & Hall.

[21] Landis JR, Koch GG. The measurement of observer agreement for categorical data. Biometrics. 1997; 33: 159–174. 843571

[22] Cooper GF, Herskovits E. A Bayesian Method for the Induction of Probabilistic Networks from Data. Machine Learning. Kluwer, Boston. 1992; 9: 309–347.

[23] Friedman N, Geiger D and Goldszmidt M. Bayesian Network Classifiers. Machine Learning. Kluwer, Boston. 1997; 29: 131–163.

[24] Cheng J, Greiner R. Learning Bayesian Belief Network Classifiers: Algorithms and System. Proc. 14th Canadian Conference on Artificial Intelligence; 2001.

[25] Madden MG. On the Classification Performance of TAN and General Bayesian Networks In: Bramer M., Petridis M., Coenen F. (eds) Research and Development in Intelligent Systems XXV. SGAI 2008. Springer, London; 2009.

[26] Subbalakshmi G, Ramesh K, Rao MC. Decision support in heart disease prediction system using naive Bayes. Indian J Comput Sci Eng. 2011; 2:170–6.

[27] Molnar C. Interpretable Machine Learning. Retrieved from https://christophm.github.io/interpretable-ml-book/. 2018.

[28] Miller T. Explanation in Artificial Intelligence: Insights from the Social Sciences. arXiv Preprint arXiv: 1706.07269. 2017.

[29] Ribeiro MT, Singh S. Guestrin C. "Why Should I Trust You?": Explaining the Predictions of Any Classifier. In: ACM SIGKDD International Conference on Knowledge Discovery and Data Mining (KDD); 2016.

[30] Lowd D, Domingos P. Naive Bayes Models for Probability Estimation. Proceedings of the 22nd International Conferenceon Machine Learning, August 7–11, Bonn, Germany, ACM; 2005. 529–536.

[31] Heckerman D. Bayesian networks for data mining. Data mining and knowledge discovery; 1997 1(1):79–119.

[32] Heckerman D, Geiger D, Chickering DM. Learning Bayesian networks: the combination of knowledge and statistical data. Machine Learning; 1995 20:197–243.

[33] Cooper GF. The computational complexity of probabilistic inference using Bayesian belief networks. Artificial Intelligence; 1990 42(2–3):393–405.

[34] Maimon O, Rokach L. Data Mining and Knowledge Discovery. Springer Science and Business Media; 2005.

[35] Mohan V. Decision Trees: A comparison of various algorithms for building Decision Trees. http://cs.jhu.edu/~vmohan3/document/ai_dt.pdf.

[36] Stern MK, Beck JE, Woolf BP. Naïve Bayes Classifiers for User Modeling. Center for Knowledge Communication, Computer Science Department, University of Massachusetts. 1999.

[37] Groza T, Köhler S, Moldenhauer D, Vasilevsky N, Baynam G, Zemojtel T, Schriml LM, Kibbe WA, Schofield PN, Beck T, Vasant D. The human phenotype ontology: semantic unification of common and rare disease. The American Journal of Human Genetics. 2015; 97:111–24. 10.1016/j.ajhg.2015.05.020 26119816 PMC4572507

[38] Shen F, Liu S, Wang Y, Wang Y, Wang L, Afzal N, Liu H. Leveraging collaborative filtering to accelerate rare disease diagnosis. AMIA Annual Symposium Proceedings. 2017; 1554–1563. PMC5977716 29854225

[39] Johnson BA, van Diggelen OP, Dajnoki A, Bodamer OA. Diagnosing lysosomal storage disorders: mucopolysaccharidosis type II. Curr Protoc Hum Genet. 2013; 79:17.14. 10.1002/0471142905.hg1714s79 24510650

